# Non-union scaphoid: Four-corner fusion of the wrist

**DOI:** 10.4103/0019-5413.61908

**Published:** 2010

**Authors:** Ravi K Gupta, Dalvir S Chauhan, Harmeet Singh

**Affiliations:** Department of Orthopaedics, Government Medical College Hospital, Chandigarh, India

**Keywords:** Four corner fusion, Cervical H-plate, nonunion scaphoid

## Abstract

**Background:**

Four-corner fusion of the wrist is an option for management of non-union scaphoid with painful arthritis of the wrist. Various surgical techniques have been devised for four-corner fusion, with inconsistent results. We present our experience of four-corner fusion achieved using a standard H-plate, designed originally for anterior cervical plating.

**Materials and Methods:**

The study is a retrospective analysis of six cases of painful wrist arthritis resulting from nonunion of scaphoid treated by four-corner fusion, between 1996 and 2004. The average duration of follow-up was 5.8 years. Each patient was evaluated clinically according to the rating scales described by Bach.

**Results:**

The mean grip-strength calculated as a percentage of the uninvolved side was 47% pre-operatively, and 74% post- operatively at the final follow-up. The difference between the preoperative and postoperative ‘pain ratings’ and ‘activity ratings’ was found to be statistically significant (*P*<0.001). Mean time to fusion was 16.1 weeks. Dorsal impingement was the most common associated problem.

**Conclusions:**

H-plate, used for four-corner fusion, provides rigid fixation, ensures fusion, and is a good alternative to the available options.

## INTRODUCTION

Untreated scaphoid non-union leads to arthritis accompanied by pain, weakness, and restriction of wrist motion.^1^ Total wrist arthrodesis provides predictable pain relief in arthritic conditions of the wrist, but at the cost of elimination of all radio-carpal and inter- carpal motion.^2^ Procedures that preserve some motion while providing pain-relief are therefore preferable. Two of the motion-preserving procedures commonly performed are proximal row carpectomy and four-corner arthrodesis.^3^,^4^ Proximal row carpectomy provides similar grip strength and better range of motion, and is technically less demanding in comparison to limited arthrodesis by four-corner fusion.^5^ However, it is not useful when associated capito-lunate arthritis is present. Secondly, it has a long-term risk of developing radio-carpal arthritis.^5^ Although consensus regarding ideal motion-preserving surgical procedure for painful arthritic wrist is lacking, Watson strongly advised limited arthrodesis with scaphoid excision.^6^

Various surgical techniques have been devised for four- corner fusion. Previously, four-corner arthrodesis has been attempted using staples or K-wires for fixation, but a less reliable fixation sometimes resulted in risk of failure of fusion.^7^ More recently, implants such as spider plate and differential pitch cannulated screws have been used to achieve rigid fixation, with variable results.^8^,^9^ In a recent study, a high rate of non-union was reported using the spider plate, with fusion being achieved in only 16.67% of the cases.^9^

We present this study of achieving four-corner fusions by using a standard H-plate, designed originally for anterior cervical plating.

## MATERIALS AND METHODS

This study is a retrospective follow-up analysis of six cases with painful arthritis of the wrist secondary to non-union of the scaphoid, treated by four corner fusions between 1996 and 2004 at our institute. None of the patients included in the study had undergone any previous surgical procedure on the wrist. Three patients were managed by prolonged immobilization; one by repeated massage around wrist and the remaining two did not take treatment before presenting.

The average time between injury and presentation was 3.27 years (1.2 to 4.82 years). The average duration of follow-up was 5.8 years (range 2.8 years-10 years) [Table 1].

**Table 1:** Clinical details of the patients

Age	Sex	Side	Side	Duration since injury (years)	Occupation
27	M	D	R	1.2	Cricket player
38	M	ND	L	3.80	Laborer
49	M	D	R	2	Computer engineer
43	F	ND	L	3.62	Housewife
24	M	D	R	4.82	Student
32	M	D	R	4.20	Laborer

D = Dominant, ND = Non dominant, R = Right, L = Left

Each patient was evaluated clinically pre-operatively and assigned a ‘pain rating’ and ‘activity rating’, as per the rating scale described by Bach^10^ [Table 2].

**Table 2:** Bach's pain and activity rating scale^10^

Pain rating scale from 1 to 6
No pain
Pain with heavy labor but tolerable
Pain with moderate labor
Pain with light labor of work activities
Pain present with all the activities, relieved with rest
Pain present at all times
Activity rating scale from 1 to 5
No limitations, able to do heavy labor, strenuous sports
Some limitations with heavy labor
Limited to moderate work activities
Limited to light duty work
Unable to perform normal activities of daily living

### Operative procedure

**Figure 1:**
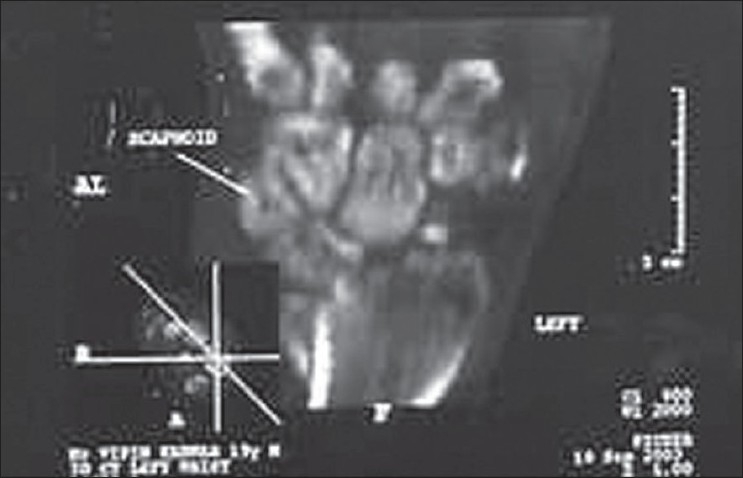
Preoperative CT scan showing nonunion fractured scaphoid with SLAC wrist

**Figure 2:**
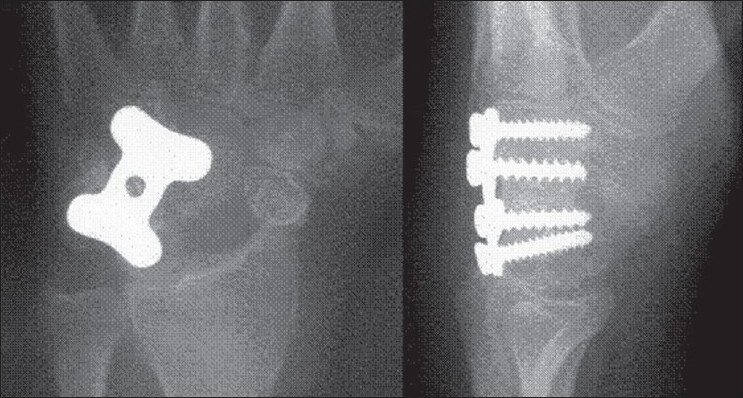
Antero-posterior and lateral radiograph showing four-corner fusion, with H-plate in place

All surgeries were performed under general anesthesia, with the patient supine, and with a pneumatic tourniquet applied to obtain a clear field. Through a longitudinal incision dorsally, the third dorsal compartment was opened. The extensor pollicis tendon was retracted laterally, and the wrist extensors retracted medially. The dorsal capsule was opened, scaphoid excised, and articular surfaces between lunate, triquetral, capitate and hamate denuded of the hyaline cartilage. Cortico-cancellous dowel of bone graft harvested from the iliac crest was impacted in the center of the four bones, and additional cancellous bone was packed between the denuded surfaces of the bones. After aligning the bones into anatomical position, with special attention to maintain a neutral position of the lunate, fixation was achieved using 2.7 mm fully threaded cancellous screws inserted one into each bone through the H-plate. [Figures 1 and 2] Post-operatively, a removable splint was applied, usually for 3-4 weeks.

## RESULTS

Average age was 35.5 years (range 27 to 49 years). The four injuries were to dominant side. The mean pre-operative and postoperative ranges of motion at the wrist are listed in Table 3.

**Table 3:** Preoperative and postoperative mean range of motion

	Pre-op range of motion (degrees)	Post-op range of motion (degrees)
Flexion	38	26
Extension	27	16
Radial deviation	5	4
Ulnar deviation	18	14

Pre-op = Preoperative, Post-op = Postoperative

The mean grip-strength calculated as a percentage of the uninvolved side was 47% pre-operatively, and 74% postoperatively at final follow-up.

The pre-operative and postoperative Bach scores for the study population are listed in Table 4, with the mean pre-operative pain rating being 5.66, and the mean postoperative pain rating being 2.16. The mean preoperative activity rating was 5, while the mean postoperative activity rating was 2.2.

**Table 4:** Results based on Bach scores

Sr. No.	Pain rating		Activity rating	
		
	Pre-op	Final post-op	Pre-op	Final post-op
1	6	2	5	2
2	6	3	5	3
3	6	2	5	2
4	5	2	5	1
5	5	2	5	1
6	6	2	5	2
Mean	5.66	2.16	5	2.2

Pre-op = Preoperative, Post-op = Postoperative

These scores were compared statistically using the paired t-test, and the difference between the pre-operative and postoperative ‘pain ratings’ (*P*<0.001) and ‘activity ratings’ (*P*<0.001) was found to be statistically significant. Mean time to fusion was 16.1 weeks. In one of the patient, the fusion was delayed up to 22 weeks

## DISCUSSION

Cervical H-plate is universally available implant. Being a relatively rigid method of fixation, it ensures fusion more reliably as compared to staples and K-wires, and the spider plate. In our series satisfactory fusion and pain relief was achieved in all the patients. The amount of wrist motion (total arc) obtained (42% of the uninvolved side) and grip strength (more than 70% of the uninvolved side) was acceptable to the patients.

A number of authors have shown satisfactory results of four-corner fusion performed as a salvage of non union scaphoid.^4^,^5^,^11^,^12^ Our results are comparable to those studies corroborating the fact that, with proper indication, the procedure of four corner fusion gives optimum results.

El-Mowafi *et al*. successfully achieved the four-corner fusion by using K wires with further immobilization of the wrist with cast.^12^ Garcia-López *et al*. achieved four-corner fusion by using the screw fixation.^13^ In order to prevent the collapse of the fused bones, however, they filled the void of the excised scaphoid by using the anchovy of extensor carpi radialis longus tendon.^13^ Chung *et al*.^8^ used a spider web plate for stabilization and concluded that four-corner fusion using the first-generation Spider plate technique had the advantage of earlier mobility and more patient comfort from absence of protruding Kirschner wires; however, patients continued to have disabling pain, functional limitations, work impairment, and low satisfaction scores postoperatively. Commenting on the failures seen in 3 of the 11 of their patients, they also felt the need for a better implant designed to avoid implant failure. In a comparative study of using traditional implants (K wires, screws and staples) and the circular plate, Vance *et al*.^14^ observed 26% nonunion with loose hardware in the plate group compared with 3% in the traditional group and 22% hardware impingement in the plate group compared with 3% in the traditional group. They concluded that the increased complication and dissatisfaction rates associated with plate fixation was attributable to possible biomechanical imperfections or increased technical demands with the circular plates.

The procedure of four-corner fusion results in loss of motion at the wrist almost to the tune of 50%, which is significant. However, the other options of proximal row carpectomy and total wrist fusion are relatively inferior options as compared to the four corner fusion with the former one having a long term risk of wrist arthritis and the latter one with disadvantage of total loss of motion.^2^,^5^

The usual complications of donor site morbidity in case of harvesting of graft from the iliac crest were not seen in any of our patients probably due to a small amount of graft required for the procedure. We feel ‘H’ plate, originally designed for the stabilization of cervical vertebral bodies is able to tolerate significant loads. We did not find any of the problems related to tendon impingement, due to the low profile design of the plate. Further, as per our per- operative observations, the four holes in the four limbs of the ‘H’ have almost exact fit onto the bodies of the four bones constituting four corners (lunate, capitate, hamate, triquetrum). The central hole in the ‘H’ can be additionally used for passing an additional screw into the dowel graft placed in the center of the four corners; however, we have not used it in any of our patients.

All the patients in our series successfully achieved fusion without any failure of the implant. The functional scores seen in all of our patients were also satisfactory, thus implying that ‘H’ plate may prove to be a reasonable alternative to the traditional implants like K wires, screws or spider plate.

The low cost stainless steel indigenously produced ‘H’ plate is likely to make it a favored implant for the surgeons working in developing countries. However, the sample size of our study being small, further larger and controlled multicentric studies are required to know the more detailed results of this technique.
